# Use of Clinical Notes to Assess Neuropsychiatric Events After Montelukast Initiation

**DOI:** 10.1001/jamanetworkopen.2025.58433

**Published:** 2026-02-12

**Authors:** Dena H. Jaffe, Elise Berliner, Bridget L. Balkaran, Austin Yue, Kyla Finlayson, Olga Guijon, Michael M. Chu, Louis Ehwerhemuepha, Lior Seluk, Michael E. Wechsler, Sengwee Toh, Jenna Wong, Kimberly J. Dandreo, Rishi J. Desai, Sarah K. Dutcher, Jummai Apata, Jamal T. Jones, Yong Ma, Jie (Jenni) Li

**Affiliations:** 1 Oracle Health, Petah Tikva, Israel; 2 Oracle Life Sciences, Kansas City, Missouri; 3 Children’s Hospital of Orange County, Orange, California; 4 Department of Pediatrics, University of California, Irvine; 5 National Jewish Health, Denver, Colorado; 6 Department of Population Medicine, Harvard Pilgrim Health Care Institute, Harvard Medical School, Boston, Massachusetts; 7 Division of Pharmacoepidemiology and Pharmacoeconomics, Department of Medicine, Brigham and Women’s Hospital and Harvard Medical School, Boston, Massachusetts; 8 Center for Drug Evaluation and Research, US Food and Drug Administration, Silver Spring, Maryland

## Abstract

**Importance:**

Prior drug safety studies investigating the risk of neuropsychiatric events (NPEs) after montelukast initiation have been limited by methodological issues, including incomplete confounder control and unmeasured outcomes associated with completeness of structured data, as well as events recorded only in unstructured clinical notes.

**Objective:**

To examine the value associated with using linked claims and electronic health records (EHRs) (structured and unstructured data) while investigating the risk of any NPE among patients with asthma initiating montelukast compared with inhaled corticosteroids (ICSs).

**Design, Setting, and Participants:**

This retrospective cohort study used Oracle EHR Real-World Data linked to a national US claims dataset (July 1, 2015, to June 30, 2022) to identify patients aged 6 to 80 years with asthma newly initiating montelukast or ICSs. The data were analyzed between December 13, 2022, and August 2, 2024.

**Main Outcomes and Measures:**

The primary outcome of any NPE and covariates was assessed using the incremental addition of each data source: analysis 1, claims-only data; analysis 2, claims plus structured EHR data; and analysis 3, claims plus structured and unstructured EHR data. Cox proportional hazard regression models using propensity score–matched cohorts across data sources were used to examine the risk of any NPE by treatment initiation group.

**Results:**

Among 109 076 patients (mean [SD] age at treatment initiation, 28.8 [20.5] years, 59.4% female), 39 665 (36.4%) initiated montelukast and 69 411 (63.6%) ICSs. Incidence rates per 100 person-years of the first postindex NPE increased with additional data sources for both montelukast and ICS users (analysis 1, 17.11 [95% CI, 16.86-17.36] vs 15.57 [95% CI, 15.34-15.80], respectively; analysis 2, 19.10 [95% CI, 18.83-19.38] vs 18.23 [95% CI, 17.97-18.50], respectively; analysis 3, 27.78 [95% CI, 27.43-28.13] vs 27.40 [95% CI, 27.06-27.75], respectively). Hazard ratios were attenuated across contributing data sources for analysis 1 (1.08 [95% CI, 1.05-1.11]), analysis 2 (1.04 [95% CI, 1.01-1.06]), and analysis 3 (1.01 [95% CI, 1.00-1.03]).

**Conclusions and Relevance:**

This cohort study found that clinical information from linked claims and structured and unstructured EHR data was associated with enriched measurement of patient and disease characteristics and enhanced completeness of evidence vs claims data alone. The findings, however, did not differ substantially across the incrementally contributing data sources or from prior studies. Drug safety and effectiveness studies should integrate information using clinical notes from EHRs with consideration of potential limitations, challenges, and necessary validation processes.

## Key Points

**Question:**

What is the value of including information extracted from unstructured data in a drug safety study on the association of montelukast initiation and risk of neuropsychiatric events?

**Findings:**

In this cohort study of 109 076 patients, 20% more patients with outcomes associated with neuropsychiatric events were identified when using clinical notes compared with structured data alone.

**Meaning:**

These findings suggest that drug safety studies able to use all available health care data sources, including claims and with both structured and unstructured electronic health care data, may provide more complete and granular ascertainment of study variables and lead to more robust and precise findings.

## Teaser

This cohort study examines the value associated with using structured and unstructured clinical notes linked with claims data while investigating the risk of neuropsychiatric disorders among montelukast users.

## Introduction

Drug safety surveillance relies on numerous data sources to identify, understand, and evaluate adverse reactions, events, and errors associated with medication use. The US Food and Drug Administration’s (FDA’s) Center for Drug Evaluation and Research monitors these approved medications using postmarket drug safety surveillance systems, such as the FDA Adverse Event Reporting System and Sentinel System.^1^

The Sentinel System, including its postmarket active risk identification and analysis system^2^ uses administrative claims and electronic health record (EHR) data from a distributed network of data partners.^3,4^ In Sentinel, distributed analytic programs analyze data from multiple health care sources using novel analytic tools to address methodological limitations of administrative and health care data and provide critical insights regarding medication safety to inform regulatory decision making.^5,6,7,8,9,10,11^ In 2021, Sansing-Foster et al^12^ conducted a retrospective cohort study of 914 754 patients with asthma using Sentinel’s claims data to examine concerns associated with behavioral changes and suicidality following montelukast exposure. The authors reported a decreased risk of outpatient depression and no increase in risk of inpatient depression or self-harm, noting limitations of incomplete confounder control related to patients’ sociodemographic and health characteristics and unmeasured outcomes, including mental health symptoms.^13^ Although the results of Sansing-Foster et al^12^ and other studies^14,15,16,17,18,19,20,21^ were equivocal, the FDA’s 2020 reevaluation of pharmacoepidemiology studies, case reports, and animal studies concluded with a boxed warning on the montelukast label for serious neuropsychiatric events (NPEs), such as suicidal thoughts and mood-related changes.^22,23,24,25^

To address frequently noted limitations of claims-based studies regarding the unavailability of granular clinical information to define study outcomes or confounding variables and the emergence of EHR data for filling these gaps, we designed the Multi-Source Observational Safety Study for Advanced Information Classification Using Natural Language Processing (MOSAIC-NLP) with the objective of examining the outcomes associated with adding structured and unstructured data from EHRs to traditional claims-only studies. Specifically, we examined the association between montelukast initiation and adverse NPEs for patients with asthma compared with initiation of inhaled corticosteroids (ICSs).

## Methods

This cohort study was a public health surveillance activity conducted under the authority of the FDA and, accordingly, not subject to institutional review board oversight per the Department of Health and Human Services policy for the protection of human research participants^26^ and informed consent in compliance with the Health Insurance Portability and Accountability Act and Common Rule.^27^ The study followed the Strengthening the Reporting of Observational Studies in Epidemiology (STROBE) reporting guideline.

### Study Design

This retrospective cohort study used the methodological foundation of Sansing-Foster et al^12^ (eFigure in Supplement 1).^28^ Notable differences between Sansing-Foster et al^12^ and this study included data sources (claims vs claims plus structured and unstructured EHR data), period (2000-2015 vs 2015-2022), outcomes (inpatient depressive disorders, treated outpatient depression, inpatient self-harm, and suicide vs any NPE listed in the boxed warning,^22^ excluding suicide), and granularity in covariates used for confounding adjustment (asthma severity: health care use vs health care use plus diagnoses, clinical tests, and clinical notes).

### Data Sources and Study Population

This study used the Oracle Health Real-World Data EHR^29,30,31^ linked to a national US claims dataset (Inovalon Insights, LLC) to access structured and unstructured health care-related data from 2015 to 2022 (eMethods in Supplement 1). The cohort included patients with asthma newly initiating montelukast or ICS therapy between July 1, 2015, and June 30, 2022. The overall study design considered both claims and EHR data in cohort identification (eFigure in Supplement 1).^28^ The included patients had records in both structured EHR and claims datasets, had at least 1 valid note (neither null nor scanned documents), and were aged 6 to 80 years upon initiation of montelukast or ICS monotherapy as identified using National Drug Codes in pharmacy dispensing claims. Medication initiation was defined as the study index date. Eligibility during the study period was considered as having continuous enrollment in both medical and prescription claims data for at least 6 months prior to index (with ≤45-day gap) and an asthma diagnosis of 6 months or less prior to index in claims data or any time prior to the index date in the structured EHR data. The latter criteria did not require a recorded date of occurrence as diagnoses in the EHR are not always documented in an encounter. Excluded patients had 6 months or less of prior exposure to montelukast, ICSs, long-acting β-agonists, leukotriene receptor antagonists, zafirlukast, or zileuton or same-day dispensing for both montelukast and ICS. Continuous exposures were defined using outpatient pharmacy dispensing data with an allowed gap of 30 days or less between dispensations. The first treatment episode meeting these requirements for each patient was analyzed.

### Measures and Follow-Up

Measures were identified from claims and structured and unstructured EHR data (eTable 1 in Supplement 1). Structured data used diagnosis and treatment codes and hospital use to evaluate symptoms and events associated with exposure for covariates and outcomes.^28^ Unstructured clinical notes obtained from natural language processing (NLP) models augmented the structured data.^32^ Validation of the NLP process was performed during model training using quantitative performance metrics (accuracy, precision, recall, and F1 scores) and qualitative evaluations of 600 clinical notes by subject matter experts comparing the extracted vs manually labeled entities from the notes (E. Berliner, PhD, unpublished data, September 2025). Entities with confidence scores of at least 0.5, indicating model certainty to correctly classify (vs manual annotation) the textual mention, were derived using a bidirectional long short-term memory network-conditional random field model (range, 0-1) and integrated into the study dataset.^33^ Assertion status (present, past, or family history) of the entity, site of care, and date of the clinical note relative to the patient’s index date were also considered for covariate (preindex) or outcome (postindex) designation.^28,32^

Any NPE was the primary outcome of interest defined using diagnoses, medications dispensed, symptoms, health care use, or reports in clinical notes based on Sansing-Foster et al^12^ and the FDA’s boxed warning for montelukast.^22^ Secondary outcomes, including sleep-related and psychiatric-related events, were components of the composite outcome of any NPE. Sleep events included circadian rhythm disorder, parasomnia, movement disorder, undefined sleep disorders, sleepwalking, and trouble sleeping. Psychiatric outcomes included adult personality disorder, agitation, anxiety, attention difficulties (eg, attention-deficit/hyperactivity disorder, hyperactivity, aggression), confusion or disorientation, delusions, hallucinations, irritability, memory problems, mood disorders (ie, depressive, bipolar or mania, other mood disorders), obsessive-compulsive disorder, psychotic disorder, restlessness, self-harm, tremor or shakiness, and uncontrolled muscle movement. Covariates were identified within 6 months prior to the index date regardless of data source, including sociodemographic characteristics (age at treatment initiation, sex, race [American Indian or Alaska Native, Asian, or Native Hawaiian or Pacific Islander (collapsed into 1 category due to low numbers); Black or African American; White; or other (multiracial or included ethnicity as race)], ethnicity [Hispanic or Latino or non-Hispanic or Latino]), clinical and family history of conditions and symptoms, health care resource use, past prescription drug dispensing, asthma-related characteristics, and lifestyle characteristics. Race and ethnicity were identified from the EHR and included as covariates in this analysis as the burden of disease, access to care, response to treatment, and outcomes differ by these groups.^34^ Follow-up began on the first day after exposure initiation and continued until the first occurrence of any of the following: treatment crossover; dispensing of an oral corticosteroid, a long-acting β-agonist, or a leukotriene receptor antagonist; any NPE; treatment cessation; death; or study end date.

### Statistical Analysis

Three analytic approaches were used to examine the value of incrementally contributing sources of covariate and outcome data. Analysis 1 included claims data only (similar to Sansing-Foster et al^12^). Analysis 2 included claims plus structured EHR data. Analysis 3 included claims plus structured and unstructured EHR data. Data were analyzed between December 13, 2022, and August 2, 2024, using R, version 4.1 (R Foundation for Statistical Computing).

Baseline patient characteristics were reported using descriptive statistics. Propensity scores were generated for each analysis using logistic regression models estimating the probability of initiating montelukast conditional on the previously described covariates to account for measured confounding.^35^ Montelukast initiators were matched to ICS initiators on the calculated propensity score in a 1-to-1 ratio using the nearest neighbor matching algorithm with 0.05 caliper, similar to Sansing-Foster et al.^12^ Standardized mean differences were used to assess covariate balance for each analysis.

Rates of the first NPE following index (incidence rates [IRs] per 100 person-years) were calculated for the composite outcome of any NPE among propensity score–matched cohorts at maximum follow-up. Cox proportional hazards regression modeling was conducted for matched conditional and unconditional cohorts and reported as hazard ratios (HRs) with 95% CIs; the latter models were presented since results were similar and to align with Sansing-Foster et al.^12^ Confidence limit ratios measuring precision were calculated.^36^ Statistical significance was set at *P* < .05.

Stratified analyses were used to examine the effect of age at index (6-11 years, 12-17 years, 18-80 years), sex (female or male), and history of psychiatric conditions (yes or no) on the association between montelukast and ICS exposure for analysis 3. Patients from the matched population were a subset within each stratum based on their original propensity scores.

Two sensitivity analyses were conducted for analysis 3. First, 39.5% of patients had no relevant clinical notes (ie, no study-relevant entities regardless of assertion status). To examine this association of no relevant notes with the effect of treatment initiation, an indicator variable representing the presence of any relevant clinical note was used in the confounding adjustment set in an additional model. Second, the risk of montelukast initiation compared with ICS was restricted to similar outcomes as used in Sansing-Foster et al.^12^

## Results

In the linked claims-EHR cohort of 109 076 patients with asthma (mean [SD] age, 28.8 [20.5] years; 59.4% female and 40.6% male; 3.1% identified as American Indian or Alaska Native, Asian, or Native Hawaiian or Pacific Islander; 21.4% as Black or African American; and 54.7% as White race; 24.9% identified as Hispanic or Latino and 67.1% as non-Hispanic or Latino ethnicity; 27.4% with commercial insurance and 66.0% with Medicaid), 39 665 (36.4%) initiated montelukast and 69 411 (63.6%) initiated ICSs (eTable 2 in Supplement 1). Most patients (81.9%) were identified between the study years 2015 and 2019.

Table 1 presents the distribution of covariates from each propensity score–matched analysis. Across analytic groups, matched cohorts retained most montelukast initiators (analysis 1, 95.8%; analysis 2, 90.3%; analysis 3, 89.8%). Demographic characteristics of matched patients receiving montelukast were similar to unmatched baseline characteristics (difference, <2 percentage points). Across analytic groups 1 to 3, patients were a mean (SD) age of 29.1 (20.9) years, 29.7 (20.7) years, and 29.8 (20.8) years, respectively; 61.2%, 61.0%, and 61.0% were female, respectively; and 62.1%, 62.5%, and 62.4% were Medicaid insured. For current health status across analytic groups 1 to 3, respectively, 5.9%, 6.3%, and 7.8% of patients had a prior diagnosis of chronic obstructive pulmonary disease (COPD); 18.9%, 24.7%, and 30.2% had allergic rhinitis; and 65.4%, 67.0%, and 67.0% had an other respiratory disorder (eg, influenza, acute bronchitis). Covariates, including prescription medication use and specific comorbidities (eg, attention difficulties, mood disorders, gastroesophageal reflux disease, ischemic heart disease, overweight or obesity, dermatitis or eczema, type 2 diabetes, or chronic obstructive pulmonary disease) were captured at consistent rates across the 3 analytic approaches using different data sources.

**Table 1.** Covariate Distribution Identified Within 6 Months Before Index in Matched Cohorts From the MOSAIC-NLP

Covariate^a^	Patients, No. (%)					
	Analysis 1 (claims data only)		Analysis 2 (claims plus structured EHR data)		Analysis 3 (claims plus structured and unstructured EHR data)	
	Montelukast (n = 38 008)	ICS (n = 38 008)	Montelukast (n = 35 810)	ICS (n = 35 810)	Montelukast (n = 35 622)	ICS (n = 35 622)
**Demographics**						
Age at treatment initiation (index), mean (SD), y	29.9 (20.4)	29.5 (21.4)	29.8 (20.4)	29.6 (21.1)	29.9 (20.4)	29.7 (21.2)
Sex						
Female	23 398 (61.6)	23 125 (60.8)	21 907 (61.2)	21 778 (60.8)	21 798 (61.2)	21 686 (60.9)
Male	14 610 (38.4)	14 883 (39.2)	13 903 (38.8)	14 032 (39.2)	13 903 (38.8)	14 032 (39.2)
Married or living with a partner	NA	NA	6986 (19.5)	6839 (19.1)	6908 (19.4)	6919 (19.4)
Race						
American Indian or Alaska Native, Asian, or Native Hawaiian or Pacific Islander	NA	NA	1074 (3.0)	1101 (3.1)	1066 (3.0)	1054 (3.0)
Black or African American	NA	NA	7202 (20.1)	7242 (20.2)	7156 (20.1)	7182 (20.2)
White	NA	NA	20 767 (58.0)	20 559 (57.4)	20 659 (58.0)	20 682 (58.1)
Other^b^	NA	NA	4535 (12.7)	4634 (12.9)	4513 (12.7)	4504 (12.6)
Missing	NA	NA	2232 (6.2)	2274 (6.4)	2228 (6.3)	2200 (6.2)
Ethnicity						
Hispanic or Latino	NA	NA	8593 (24.0)	8798 (24.6)	8545 (24.0)	8703 (24.4)
Non-Hispanic or Latino	NA	NA	24 583 (68.7)	24 187 (67.5)	24 440 (68.6)	24 155 (67.8)
Missing	NA	NA	2634 (7.4)	2825 (7.9)	2637 (7.4)	2764 (7.8)
Insurance status						
Commercial	11 661 (30.7)	11 536 (30.4)	10 916 (30.5)	10 676 (29.8)	10 852 (30.5)	10 713 (30.1)
Medicaid	23 531 (61.9)	23 703 (62.4)	22 281 (62.2)	22 512 (62.9)	22 181 (62.3)	22 303 (62.6)
Medicare	2302 (6.1)	2277 (6.0)	2127 (5.9)	2127 (5.9)	2112 (5.9)	2113 (5.9)
Other or missing^c^	514 (1.4)	492 (1.3)	486 (1.4)	495 (1.4)	477 (1.3)	493 (1.4)
Region						
Midwest	NA	NA	5067 (14.2)	5016 (14.0)	5027 (14.1)	5027 (14.1)
Northeast	NA	NA	6116 (17.1)	6015 (16.8)	6081 (17.1)	6041 (17.0)
South	NA	NA	11 634 (32.5)	11 546 (32.2)	11 582 (32.5)	11 596 (32.6)
West	NA	NA	12 814 (35.8)	13 046 (36.4)	12 752 (35.8)	12 785 (35.9)
Missing	NA	NA	179 (0.5)	187 (0.5)	180 (0.5)	173 (0.5)
Health care systems, No.	NA	NA	112	112	111	110
**Current health status**						
CCI, mean (SD)^d^	0.6 (1.2)	0.5 (1.2)	0.8 (1.4)	0.8 (1.3)	0.8 (1.4)	0.8 (1.3)
Respiratory disorders, other^e^	25 762 (67.8)	25 688 (67.6)	24 668 (68.9)	24 660 (68.9)	24 541 (68.9)	24 507 (68.8)
Attention difficulties^f^	11 956 (31.5)	11 717 (30.8)	11 204 (31.3)	11 137 (31.1)	11 536 (32.4)	11 477 (32.2)
Cough	10 056 (26.5)	9999 (26.3)	10 275 (28.7)	10 361 (28.9)	12 992 (36.5)	12 974 (36.4)
Allergic rhinitis	8819 (23.2)	8867 (23.3)	10 468 (29.2)	10 245 (28.6)	10 787 (30.3)	10 773 (30.2)
Anxiety^g^	6273 (16.5)	6121 (16.1)	7656 (21.4)	7616 (21.3)	8536 (24.0)	8375 (23.5)
Mood disorder						
Any	5597 (14.7)	5453 (14.4)	5303 (14.8)	5276 (14.7)	5335 (15.0)	5287 (14.8)
Depression	3497 (9.2)	3438 (9.1)	4976 (13.9)	4955 (13.8)	5889 (16.5)	5857 (16.4)
Bipolar disorder or manic episode	989 (2.6)	923 (2.4)	1336 (3.7)	1351 (3.8)	1339 (3.8)	1283 (3.6)
GERD	5397 (14.2)	4751 (12.5)	5240 (14.6)	4841 (13.5)	5208 (14.6)	4785 (13.4)
Ischemic heart disease	779 (2.1)	844 (2.2)	722 (2.0)	789 (2.2)	724 (2.0)	787 (2.2)
Overweight or obesity	4575 (12.0)	4484 (11.8)	5545 (15.5)	5491 (15.3)	5498 (15.4)	5439 (15.3)
Dermatitis and eczema	3512 (9.2)	3497 (9.2)	3727 (10.4)	3557 (9.9)	3669 (10.3)	3546 (10.0)
Type 2 diabetes	3392 (8.9)	3410 (9.0)	3374 (9.4)	3331 (9.3)	3347 (9.4)	3354 (9.4)
COPD	2126 (5.6)	2048 (5.4)	2103 (5.9)	2123 (5.9)	2219 (6.2)	2210 (6.2)
Psychotic disorder	589 (1.6)	582 (1.5)	965 (2.7)	978 (2.7)	980 (2.8)	968 (2.7)
Sleep disorder^h^	526 (1.4)	521 (1.4)	927 (2.6)	931 (2.6)	2327 (6.5)	2327 (6.5)
Substance abuse	196 (0.5)	183 (0.5)	1123 (3.1)	1185 (3.3)	1914 (5.4)	1891 (5.3)
Obsessive-compulsive disorder	9 (<0.1)	11 (<0.1)	112 (0.3)	116 (0.3)	169 (0.5)	170 (0.5)
Self-harm^i^	272 (0.7)	282 (0.7)	262 (0.7)	222 (0.6)	1012 (2.8)	1033 (2.9)
Delusions	NA	NA	NA	NA	62 (0.2)	58 (0.2)
Hallucinations	NA	NA	NA	NA	601 (1.7)	554 (1.6)
Memory problems	NA	NA	NA	NA	165 (0.5)	166 (0.5)
Irritability	NA	NA	NA	NA	301 (0.8)	330 (0.9)
Restlessness	NA	NA	NA	NA	142 (0.4)	153 (0.4)
Agitation	NA	NA	NA	NA	112 (0.3)	127 (0.4)
Confusion or disorientation	NA	NA	NA	NA	418 (1.2)	417 (1.2)
Uncontrolled muscle movement	NA	NA	NA	NA	254 (0.7)	254 (0.7)
Tremor or shakiness	NA	NA	NA	NA	21 (0.1)	20 (0.1)
**Lifestyle characteristics** ^j^						
Exercise	NA	NA	NA	NA	241 (0.7)	232 (0.7)
Alcohol use	NA	NA	NA	NA	2221 (6.2)	2226 (6.3)
Tobacco use	NA	NA	NA	NA	3300 (9.3)	3515 (9.9)
Marijuana use	NA	NA	NA	NA	396 (1.1)	403 (1.1)
**Family medical history**						
Psychiatric disorder^k^	NA	NA	NA	NA	950 (2.7)	959 (2.7)
Sleep disorder^l^	NA	NA	NA	NA	163 (0.5)	164 (0.5)
**Prescription medication use**						
ACE inhibitors	1901 (5.0)	2085 (5.5)	1795 (5.0)	2004 (5.6)	1789 (5.0)	1998 (5.6)
Analgesics	1854 (4.9)	1781 (4.7)	1766 (4.9)	1694 (4.7)	1760 (4.9)	1763 (5.0)
Anti-inflammatories	7105 (18.7)	6911 (18.2)	6685 (18.7)	6628 (18.5)	6619 (18.6)	6508 (18.3)
Antihistamines	9233 (24.3)	9366 (24.6)	8648 (24.2)	8682 (24.2)	8597 (24.1)	8596 (24.1)
Antilipemics	3729 (9.8)	3918 (10.3)	3462 (9.7)	3707 (10.4)	3475 (9.8)	3665 (10.3)
Antirheumatics	586 (1.5)	602 (1.6)	560 (1.6)	599 (1.7)	569 (1.6)	596 (1.7)
Antitussives	23 (0.1)	15 (<0.1)	21 (0.1)	13 (<0.1)	19 (0.1)	14 (<0.1)
Bronchodilators	996 (2.6)	971 (2.6)	927 (2.6)	916 (2.6)	930 (2.6)	928 (2.6)
Decongestants	207 (0.5)	186 (0.5)	198 (0.6)	197 (0.6)	203 (0.6)	213 (0.6)
Gastric and/or reflux medications	2127 (5.6)	1862 (4.9)	2015 (5.6)	1809 (5.1)	1972 (5.5)	1768 (5.0)
Glucocorticoids	12 250 (32.2)	12 176 (32.0)	11 608 (32.4)	11 648 (32.5)	11 509 (32.3)	11 468 (32.2)
Levothyroxine	1948 (5.1)	1928 (5.1)	1819 (5.1)	1813 (5.1)	1815 (5.1)	1780 (5.0)
Metformin	1892 (5.0)	1938 (5.1)	1802 (5.0)	1882 (5.3)	1776 (5.0)	1819 (5.1)
Muscle relaxers	2879 (7.6)	2746 (7.2)	2700 (7.5)	2666 (7.4)	2716 (7.6)	2700 (7.6)
Vilanterol	376 (1.0)	327 (0.9)	367 (1.0)	318 (0.9)	362 (1.0)	327 (0.9)
Antipsychotics	3765 (9.9)	3676 (9.7)	3558 (9.9)	3604 (10.1)	3598 (10.1)	3562 (10.0)
Sedatives	1699 (4.5)	1664 (4.4)	1617 (4.5)	1579 (4.4)	1622 (4.6)	1580 (4.4)
**Indicators of asthma severity and control and asthma-related characteristics**						
Severe asthma^m^	956 (2.5)	958 (2.5)	980 (2.7)	1017 (2.8)	1049 (2.9)	1092 (3.1)
ED visit for asthma	5579 (14.7)	5499 (14.5)	6629 (18.5)	6789 (19.0)	6591 (18.5)	6621 (18.6)
Hospitalization for asthma	1723 (4.5)	1653 (4.4)	2220 (6.2)	2256 (6.3)	2199 (6.2)	2231 (6.3)
Hospitalization for other respiratory conditions or viral infections	1439 (3.8)	1372 (3.6)	1581 (4.4)	1595 (4.5)	1568 (4.4)	1592 (4.5)
≥2 Outpatient visits for asthma	29 162 (76.7)	29 257 (77.0)	27 771 (77.6)	27 983 (78.1)	27 611 (77.5)	27 656 (77.6)
Oral corticosteroids						
0	35 297 (92.9)	35 412 (93.2)	33 290 (93.0)	33 361 (93.2)	33 099 (92.9)	33 157 (93.1)
1	2234 (5.88)	2145 (5.64)	2072 (5.79)	2024 (5.65)	2072 (5.82)	2033 (5.71)
2	350 (0.9)	329 (0.9)	325 (0.9)	313 (0.9)	332 (0.9)	316 (0.9)
≥3	127 (0.33)	122 (0.32)	123 (0.34)	112 (0.31)	119 (0.33)	116 (0.33)
≥2 SABAs	15 956 (42.0)	18 855 (49.6)	15 273 (42.7)	17 934 (50.1)	15 709 (44.1)	15 811 (44.4)
≥2 Anticholinergics	408 (1.1)	404 (1.1)	393 (1.1)	392 (1.1)	393 (1.1)	408 (1.2)
Shortness of breath	NA	NA	NA	NA	3471 (9.7)	3966 (11.1)
Snoring	0	0	263 (0.7)	262 (0.7)	558 (1.6)	573 (1.6)
Wheezing	NA	NA	NA	NA	2611 (7.3)	3182 (8.9)

Abbreviations: ACE, angiotensin-converting enzyme; CCI, Charlson Comorbidity Index; COPD, chronic obstructive pulmonary disease; ED, emergency department; GERD, gastroesophageal reflux disease; ICS, inhaled corticosteroid (monotherapy); MOSAIC-NLP, Multi-Source Observational Safety Study for Advanced Information Classification Using Natural Language Processing; NA, not applicable; SABA; short-acting β-agonist.

^a^ All covariates were identified as occurring or reported in the 6 months prior to index. The standardized mean difference assessing covariate balance for the propensity score matching was less than 0.035 for all analytic groups.

^b^ Included multiracial or ethnicity reported as race.

^c^ Included self-pay and no insurance.

^d^ On a scale of 0 to 24, with higher values indicating a greater burden of disease.^37^

^e^ Included influenza and acute bronchitis.

^f^ Included attention-deficit/hyperactivity disorder, hyperactivity, and aggression.

^g^ Included diagnoses or mention of anxiety disorder, anxiety, and feeling anxious.

^h^ Included insomnia, hypersomnia, circadian rhythm disorder, parasomnia, movement disorder, other undefined sleep disorders, or treatment for sleep disorders.

^i^ Included diagnoses or mention of self-harm, self-harm ideation, suicide attempt, and suicidal ideation.

^j^ Defined as entities containing mention of the use or habits (eg, practicing sports as an indicator of exercise).

^k^ Included mention in the clinical notes of anxiety, feeling anxious, depression, obsessive-compulsive disorder, or attention difficulties.

^l^ Included mention in the clinical notes of sleep disorders, dream abnormalities, and restless leg syndrome.

^m^ Defined using diagnoses, clinical test results, or reports in clinical notes.

Covariate identification increased across analyses, especially for acute and mental health–related events, such as sleep disorders (total: analysis 1, 1.4%; analysis 2, 2.6%; analysis 3, 6.5%), substance abuse (total: analysis 1, 0.5%; analysis 2, 3.2%; analysis 3, 5.3%), and obsessive-compulsive disorder (total: analysis 1, 0; analysis 2, 0.3%; analysis 3, 0.5%) (Table 1). Asthma severity and control characteristics were enhanced with descriptors of shortness of breath and wheezing identified from unstructured EHR data only and snoring examined in all 3 sources (total: analysis 1, 0; analysis 2, 0.7%; analysis 3, 1.6%).

The proportion of patients experiencing any NPE increased substantially across analytic approaches (analysis 1, 46.5%; analysis 2, 50.7%; analysis 3, 67.4%) (Table 2). Follow-up time until censoring decreased across analytic groups and especially with the addition of unstructured data (median [IQR]: analysis 1, 924 [151-1868] days; analysis 2, 839 [143-1772] days; analysis 3, 466 [81-1389] days). Enhanced identification among NPE components was observed particularly for patients with symptoms not identified in structured data and for those identified in both structured and unstructured data, such as attention difficulties (analysis 1, 2.6%; analysis 2, 5.5%; analysis 3, 12.5%) and self-harm (analysis 1, 3.9%; analysis 2, 4.6%; analysis 3, 8.9%).

**Table 2.** Outcomes Identified Using Additional Sources of Data

Outcome^a^	Analysis 1 (claims data only)			Analysis 2 (claims plus structured EHR data)			Analysis 3 (claims plus structured and unstructured EHR data)		
	Total (n = 76 016)	Montelukast (n = 38 008)	ICS (n = 38 008)	Total ( n = 71 620)	Montelukast (n = 35 810)	ICS (n = 35 810)	Total (n = 71 244)	Montelukast (n = 35 622)	ICS (n = 35 622)
Follow-up time to censoring, d									
Mean (SD)	1039.8 (877.3)	1016.5 (872.1)	1063.1 (881.8)	995.6 (863.7)	982.4 (859.2)	1008.8 (867.9)	792.7 (806.1)	783.7 (799.9)	801.8 (812.2)
Median (IQR)	924 (151-1868)	881 (143-1823)	966 (159-1904)	839 (143-1772)	819 (140-1745)	855 (147-1801)	466 (81-1389)	455 (79-1371)	476 (83-1408)
Events per person, No.									
Mean (SD)	2.5 (1.5)	2.5 (1.5)	2.5 (1.5)	2.6 (1.7)	2.7 (1.7)	2.6 (1.7)	2.6 (1.7)	2.6 (1.7)	2.6 (1.7)
Median (IQR)	2 (1-3)	2 (1-3)	2 (1-3)	2 (1-4)	2 (1-4)	2 (1-4)	2 (1-4)	2 (1-4)	2 (1-4)
Neuropsychiatric event, No. (%)									
Any	35 316 (46.5)	18 095 (47.6)	17 221 (45.3)	36 305 (50.7)	18 333 (51.2)	17 972 (50.2)	48 011 (67.4)	24 024 (67.4)	23 987 (67.3)
Any sleep disorder^b^	12 443 (16.4)	6457 (17.0)	5986 (15.7)	14 868 (20.8)	7605 (21.2)	7263 (20.3)	18 716 (26.3)	9547 (26.8)	9169 (25.7)
Any psychiatric disorder	33 196 (43.7)	16 989 (44.7)	16 207 (42.6)	33 457 (46.7)	16 914 (47.2)	16 543 (46.2)	42 907 (60.2)	21 510 (60.4)	21 397 (60.1)
Anxiety^c^	26 098 (34.3)	13 499 (35.5)	12 599 (33.1)	27 002 (37.7)	13 742 (38.4)	13 260 (37.0)	31 309 (43.9)	15 814 (44.4)	15 495 (43.5)
Mood disorder^d^	21 620 (28.4)	11 162 (29.4)	10 458 (27.5)	20 659 (28.8)	10 488 (29.3)	10 171 (28.4)	21 573 (30.3)	10 526 (29.5)	10 077 (28.3)
Depression	16 069 (21.1)	8253 (21.7)	7816 (20.6)	15 929 (22.2)	8070 (22.5)	7859 (21.9)	20 603 (28.9)	10 918 (30.6)	10 655 (29.9)
Bipolar disorder or manic episode	4108 (5.4)	2089 (5.5)	2019 (5.3)	4359 (6.1)	2183 (6.1)	2176 (6.1)	4257 (6.0)	2185 (6.1)	2072 (5.8)
Attention difficulties^e^	1962 (2.6)	953 (2.5)	1009 (2.7)	3916 (5.5)	1884 (5.3)	2032 (5.7)	8938 (12.5)	4393 (12.3)	4545 (12.8)
Adult personality disorder	1799 (2.4)	919 (2.4)	880 (2.3)	2187 (3.1)	1093 (3.1)	1094 (3.1)	2187 (3.1)	1095 (3.1)	1092 (3.1)
Psychotic disorder	2862 ( 3.8)	1362 (3.6)	1500 (3.9)	4055 (5.7)	1967 (5.5)	2088 (5.8)	4019 (5.6)	1960 (5.5)	2059 (5.8)
Self-harm^f^	2937 (3.9)	1501 (3.9)	1436 (3.8)	3280 (4.6)	1681 (4.7)	1599 (4.5)	6348 (8.9)	3149 (8.8)	3199 (9.0)
Obsessive-compulsive disorder	48 (<0.1)	28 (<0.1)	20 (<0.1)	646 (<0.1)	336 (<0.1)	310 (<0.1)	785 (1.1)	406 (1.1)	379 (1.1)
Confusion/disorientation	0	0	0	0	0	0	2103 (3.0)	960 (2.7)	1143 (3.2)
Irritability	0	0	0	0	0	0	921 (1.3)	488 (13.7)	433 (1.2)
Uncontrolled muscle movement	0	0	0	0	0	0	949 (1.3)	481 (1.4)	468 (1.3)
Restlessness	0	0	0	0	0	0	396 (0.1)	184 (<0.1)	212 (0.1)
Agitation	0	0	0	0	0	0	261 (<0.1)	112 (<0.1)	149 (<0.1)
Memory problems	0	0	0	0	0	0	412 (0.1)	222 (0.1)	190 (0.1)
Hallucinations	0	0	0	0	0	0	2304 (3.2)	1241 (3.5)	1063 (3.0)
Delusions	0	0	0	0	0	0	152 (<0.1)	77 (<0.1)	75 (<0.1)
Tremor/shakiness	0	0	0	0	0	0	62 (<0.1)	34 (<0.1)	28 (<0.1)

Abbreviations: EHR, electronic health record; ICS, inhaled corticosteroid.

^a^ All outcomes are nonmutually exclusive. Negative changes may be due to cohort composition variability between each propensity score–matched cohort.

^b^ Included insomnia, hypersomnia, circadian rhythm disorder, parasomnia, movement disorder, other undefined sleep disorders, or treatment for sleep disorders.

^c^ Included diagnoses or mention of anxiety disorder, anxiety, and feeling anxious.

^d^ Included depression, bipolar, and manic disorders.

^e^ Included attention-deficit/hyperactivity disorder, hyperactivity, and aggression.

^f^ Included self-harm, self-harm ideation, suicide attempt, and suicidal ideation.

The IRs per 100 person-years, representing the first indication of any NPE post index, showed an overall increase from analysis 1 (from 15.57 [95% CI, 15.34-15.80] for ICSs to 17.11 [95% CI, 16.86-17.36] for montelukast) to analysis 3 (from 27.40 [95% CI, 27.06-27.75] for ICSs to 27.78 [95% CI, 27.43-28.13] for montelukast) (Figure). Anxiety was the most prevalent first-identified NPE across matched therapeutic groups and analyses (range, 39.8%-49.5%), followed by mood disorders (range, 22.7%-28.8%) and sleep disorders (range, 15.9%-18.7%) (eTable 3 in Supplement 1). Unstructured data increased the identification of incident symptomatic-type outcomes, such as confusion, attention difficulties, and hallucinations by more than 20%.

**Figure.**
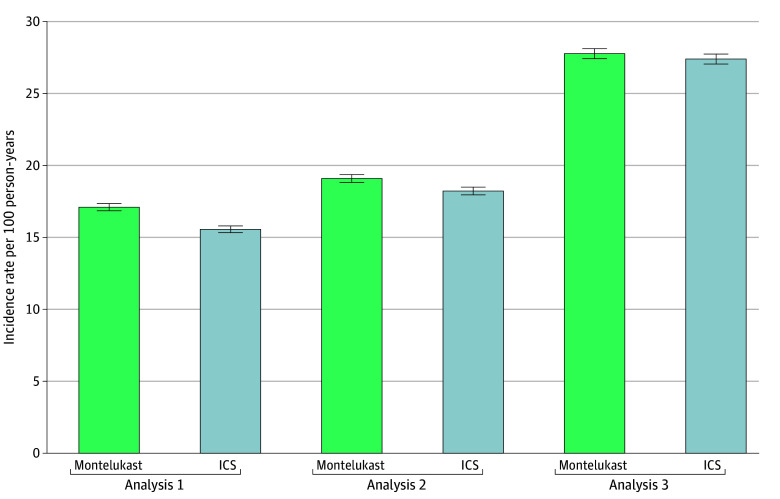
Incidence Rate Estimates for the First Neuropsychiatric Outcome in Matched Unconditional Cohorts From the Multi-Source Observational Safety Study for Advanced Information Classification Using Natural Language Processing Outcomes were identified using only claims data (analysis 1), claims plus structured electronic health record data (analysis 2), and claims plus structured and unstructured electronic health record data (analysis 3). Error bars indicate the 95% CIs. ICS indicates inhaled corticosteroid.

Unconditional matched Cox proportional hazards regression models showed a slight increased risk for primary and secondary outcomes for patients initiating montelukast compared with ICSs in analysis 1 only when adjusting for claims-based confounders but attenuated substantially when adjusting for structured and unstructured EHR-based confounders in analysis 3. Hazard ratios for the primary outcome of any NPE were as follows: analysis 1, 1.08 (95% CI, 1.05-1.11); analysis 2, 1.04 (95% CI, 1.01-1.06); and analysis 3, 1.01 (95% CI, 1.00-1.03), with narrowing confidence limit ratios (1.06, 1.05, and 1.03, respectively) (Table 3).

**Table 3.** Unconditional Matched Analysis Estimating Outcomes Associated With New Use of Montelukast Compared With Inhaled Corticosteroids Among Patients With Asthma Across the MOSAIC-NLP

Outcome	Analysis 1 (claims data only)		Analysis 2 (claims plus structured EHR data)		Analysis 3 (claims plus structured and unstructured EHR data)	
	HR (95% CI)	95% CLR	HR (95% CI)	95% CLR	HR (95% CI)	95% CLR
Neuropsychiatric event^a^	1.08 (1.05-1.11)	1.06	1.04 (1.01-1.06)	1.05	1.01 (1.00-1.03)	1.03
Sleep disorder	1.09 (1.05-1.13)	1.08	1.05 (1.02-1.09)	1.07	1.05 (1.02-1.08)	1.06
Psychiatric disorder	1.06 (1.03-1.09)	1.06	1.03 (1.01-1.06)	1.05	1.02 (1.00-1.04)	1.02

Abbreviations: CLR, confidence limit ratio; EHR, electronic health record; HR, hazard ratio; MOSAIC-NLP, Multi-Source Observational Safety Study for Advanced Information Classification Using Natural Language Processing.

^a^ Comprised both sleep disorders (insomnia, hypersomnia, circadian rhythm disorder, parasomnia, movement disorder, other undefined sleep disorders, or treatment for sleep disorders) and psychiatric disorders (anxiety, depression, mood disorder, bipolar disorder or mania, attention problems, hyperactivity, aggression, adult personality disorder, psychotic disorder, self-harm, obsessive-compulsive disorder, confusion or disorientation, irritability, uncontrolled muscle movement, restlessness, agitation, memory problems, hallucinations, delusions, tremor, or shakiness). Outcomes were mutually exclusive.

In an analysis 3 sensitivity analysis, data were separately stratified by sex, age group, and psychiatric history. Exposure to montelukast compared with ICSs was not associated with any NPE outcome except for a slight increased risk for children (aged 6-11 years) (HR, 1.05 [95% CI, 1.01-1.09]) and a decreased risk for adolescents (aged 12-17 years) (HR, 0.94 [95% CI 0.90-0.98]) (Table 4). In a sensitivity analysis assessing a differential effect of treatment exposure on any NPE for patients with and without study-relevant notes (analysis 3), the interaction term was not statistically significant (HR, 0.96 [95% CI, 0.93-1.00]; *P* = .07). Additional analyses of the risk of montelukast initiation compared with ICSs showed no association with treated depression (HR, 1.06 [95% CI, 1.02-1.10]), hospitalization for depression (HR, 0.99 [95% CI, 0.92-1.05]), and self-harm (HR, 0.98 [95% CI, 0.94-1.03]) in the analysis 3 group.

**Table 4.** Unconditional Matched Analysis Estimating Neuropsychiatric Events Associated With New Use of Montelukast Among Patients With Asthma in MOSAIC-NLP Analysis 3^a^

Patient subgroup	HR (95% CI)
Sex	
Female	1.01 (0.99-1.03)
Male	1.02 (0.99-1.05)
Age at treatment initiation (index), y	
6-11	1.05 (1.01-1.09)
12-17	0.94 (0.90-0.98)
18-80	0.98 (0.96-1.00)
Psychiatric conditions in the 6 mo prior to index	
No	1.02 (0.99-1.05)
Yes	1.01 (0.98-1.03)

Abbreviations: HR, hazard ratio; MOSAIC-NLP, Multi-Source Observational Safety Study for Advanced Information Classification Using Natural Language Processing.

^a^ Outcomes were identified in analysis 3 from claims plus structured and unstructured electronic health record data.

## Discussion

This cohort study, the MOSAIC-NLP, examined the outcomes associated with incrementally adding structured and unstructured EHR data to claims data in a drug safety study of montelukast initiation and the risk of NPEs. In this linked dataset, rates of patients with documented NPEs following treatment initiation increased moderately with the addition of structured EHR data to claims data and increased considerably with the further addition of unstructured EHR data. This incremental addition of data sources used to ascertain covariates and outcomes also contributed to the adjusted risk estimates for the association between montelukast initiation and risk of NPEs, becoming more attenuated toward the null. The magnitude of risk observed in this study aligns with those reported in two Sentinel claims-based studies,^12,38^ previous clinical trials,^15,39^ and observational studies^40,41^ reporting a minimal association between montelukast use and risk of NPEs.

Linking claims with structured and unstructured EHR data was associated with enriched patient characteristics and more comprehensive patient matching than structured data–only analyses; however, the larger variable set was associated with a reduced number of successful matches. This approach also showed an increase in outcome identification and improvement in precision around risk estimates compared with the claims-only analytic group and prior studies.^12,38,41^ Enhanced outcome identification was specifically observed for measures of mental health, for which the inclusion of unstructured data was associated with both increased detection and earlier reporting. These findings may reflect that mental health professionals treat and document milder neuropsychiatric symptoms that may not meet diagnostic criteria, while structured data are used administratively to record the primary *Diagnostic and Statistical Manual of Mental Disorders* (Fifth Edition) diagnosis for medical reimbursement.^42,43^

The lessons learned from this study lay the foundation for improving estimates of treatment effectiveness and safety in large-scale, noninterventional studies using structured and unstructured data from linked administrative and health care data sources. Natural language processing could normalize unstructured data into a structured, queryable format for analysis at scale. However, the reliability and validity of the structurized output data would be contingent upon (1) the accuracy and intent of the practitioner’s reporting in the clinical notes, which may be highly heterogeneous across practitioner types and systems; (2) the performance of the NLP model; and (3) the appropriate management of these entities for analysis.^5,32,44,45^ Regarding the latter and in contrast with the well-defined use of structured data for disease phenotyping, unstructured entity management, whereby textual mentions are operationalized into structured data for analysis, is less understood. In this study, outcomes identified from clinical notes relied on a single mention, note timing, confidence of the entity in the note, and the assertion of present. In addition, criteria might consider the number of mentions within or across notes; the note’s author and health care setting; and other contextual nuances, such as the assertion of possible, which may represent an additional trove of transient symptoms potentially relevant for safety studies. Defining and validating a framework for using clinical notes at scale and understanding the potential bias from misclassification error when using extracted entities would be a necessary next step. Here, we assessed NLP model precision (ie, positive predictive value) and recall (ie, sensitivity) against manual extractions with high model performance and confidence that these were not false-positive findings and a 2% error rate or less for entities of interest (E. Berliner, PhD, unpublished data, September 2025). To further reduce error, a reduced sensitivity was allowed to ensure consistently high positive predictive values by including present while excluding probable or possible tags as outcome events. Incorporating NLP tools upstream into EHR system design may help standardize and simplify documentation procedures across different health care settings.

### Strengths and Limitations

In this study with an active-comparator design, the linkage of 2 large population-based datasets allowed for a robust examination of the association between montelukast compared with ICSs and NPEs, accounting for numerous covariates and mitigating biases of nontreatment, outcome misclassification, and confounding due to incomplete capture of baseline risk from claims alone. A limitation of the study is its design, which may not represent the full cohort of potential patients in each individual dataset.^46^ First, linkage with claims data restricted results interpretation to an insured population only; the study population comprised 66% Medicaid recipients, in whom asthma is typically less well controlled than in privately insured individuals.^47^ Second, we included patients with clinical notes, recognizing that, similar to structured EHR data, the absence of clinical notes may indicate a nonresponse or less severe response that did not result in a health care encounter.^48^ Moreover, among patients with notes, only 61% of the cohort had relevant notes for ascertaining study measures, although a sensitivity analysis indicated no difference in the risk estimates between those with and without relevant notes. Third, information bias may occur when less severe NPEs resolve without a health care encounter due to treatment discontinuation with or without physician consultation or documentation and might be reported differentially by age group, with older patients not discussing adverse events compared with parents reporting safety concerns.^49,50,51,52^ Differential reporting by treatment group may indeed be associated with awareness and concerns specific to corticosteroid use in children or medication label warnings, such as the montelukast boxed warning.^22,53,54^ The limited duration of the preindex period may have obviated extant predispositions without recent manifestations. Finally, the scope of this study precluded additional analysis with negative or positive control outcomes or exposures to examine robustness of the study findings to unmeasured biases.

## Conclusions

This cohort study of the safety of patients with asthma initiating montelukast found that a broadened scope and scale of clinical information extracted from EHR data were associated with enriched measurement of patient and disease characteristics; minimization of residual confounding; and, ultimately, enhanced strength and accuracy of safety evidence generated from the linked data sources compared with that from claims data alone. This analysis provides insights into the challenges of linking claims data with EHRs in the context of measuring covariates and outcomes in large-scale drug safety studies and encouraging cautions in the interpretation of the study results when used for health care decision-making. The results confirm and support previous findings of no clinically meaningful association for use of montelukast for patients with asthma with respect to experiencing any neuropsychiatric outcome.

## Notes

Supplement 1. **eMethods.** Study Methodology **eTable 1.** Study Measures and Source of Data **eTable 2.** Baseline Demographics of Patients With Asthma in Claims Linked to Structured and Unstructured EHR Data **eTable 3.** Percentage Distribution of Components of Incident Neuropsychiatric Outcomes in Matched Unconditional Cohorts From the MOSAIC-NLP Analyses **eFigure.** Study Design
eReferences.

Supplement 2.
Data Sharing Statement
