# Supplementary material for: Use of Clinical Notes to Assess Neuropsychiatric Events After Montelukast Initiation
Source: JAMA Netw Open. 2026 Feb 12;9(2):e2558433. doi: 10.1001/jamanetworkopen.2025.58433 (PMC12902883; doi:10.1001/jamanetworkopen.2025.58433)
Supplement: Supplement 1. — eMethods. Study Methodology eTable 1. Study Measures and Source of Data eTable 2. Baseline Demographics of Patients With Asthma in Claims Linked to Structured and Unstructured EHR Data eTable 3. Percentage Distribution of Components of Incident Neuropsychiatric Outcomes in Matched Unconditional Cohorts From the MOSAIC-NLP Analyses eFigure. Study Design eReferences. [file jamanetwopen-e2558433-s001.pdf]

Supplemental Online Content

Jaffe DH, Berliner E, Balkaran BL, et al. Leveraging clinical notes in a safety study of neuropsychiatric events after montelukast initiation. *JAMA Netw Open*. 2026;9(2):e2558433. doi:10.1001/jamanetworkopen.2025.58433

eMethods. Study Methodology

eTable 1. Study Measures and Source of Data

eTable 2. Baseline Demographics of Patients With Asthma in Claims Linked to Structured and Unstructured EHR Data

eTable 3. Percentage Distribution of Components of Incident Neuropsychiatric Outcomes in Matched Unconditional Cohorts From the MOSAIC-NLP Analyses

eFigure 1. Study Design

eReferences

This supplemental material has been provided by the authors to give readers additional information about their work.

© 2026 Jaffe DH et al. *JAMA Network Open*.

eMethods. Study METHODOLOGY

Oracle Health Real-World Data electronic health records (OHRWD)[1,2] was linked to a national US claims data source (2015-2022). The third-party vendor Datavant[3] created tokens for linkage of each dataset with a linkage rate of 21.9%, representing the linkability of all OHRWD patients across the entire time period of the dataset starting from ~1980 with claims data with eligibility from 2015. The OHRWD is multicenter EHR database aggregating data from 120+ participating healthcare systems across the United States for 100+ million patients, 1+ billion healthcare encounters, and 3.5+ billion clinical notes representing community, critical access, academic, and teaching hospitals, and outpatient clinics.[4] Structured data included patient and healthcare system characteristics, and patient diagnostic and clinical data (**eTable 1**). Unstructured data comprised semi-structured and unstructured text notes, images, and PDFs. Datavant mortality data was integrated into the OHRWD via Datavant tokens to improve data completeness and used for censoring in the present study.[3] The United States claims dataset consists of 150+ payer sources (commercial claims, Medicare Advantage, and Medicaid data) from 50 states, DC, and Puerto Rico, and includes medical, laboratory, and pharmacy claims as well as enrolment data (Inovalon Insights, LLC; 2015-2022).

The data sources and collection procedures were validated using extensive quality control procedures, as described further below.[4,5] The OHRWD dataset is stored in the enterprise data warehouse and processed through well-documented data capabilities and quality controls to ensure a high grade of data curation for research purposes. Code lists used to identify patients, medications, and to define outcomes were verified by expert advisors and documented in the study protocol.[4,5]

Race and ethnicity were included as covariates in this analysis as the burden of disease, access to care, response to treatment, and outcomes differ by these groups.[6] Race and ethnicity were from the OHRWD that mapped source EHR data for race and ethnicity using the US Center for Disease Control and Prevention code sets.[7] In the OHRWD, six categories were reported for race: Asian, or American Indian or Alaska Native, or Native Hawaiian or Other Pacific Islander; Black or African American; White; Other; and Unknown. Other included ‘other race’ or multiple races. In the OHRWD, three categories were reported for ethnicity: Hispanic or Latino; Non-Hispanic or Latino; and Unknown.

© 2026 Jaffe DH et al. *JAMA Network Open*.

eTable 2. Study Measures and Source of Data

Variable	Measure type		Data source		
	Covariate	Outcome	Claims	EHR structured	EHR unstructured
**Demographics**					
Age	X		X		
Sex	X		X		
Race	X			X	
Ethnicity	X			X	
Region	X			X	
Marital status	X			X	
**Health status**					
Agitation	X	X			X
Allergic rhinitis	X		X	X	
Anxiety	X	X	X	X	X
Attention difficulties[a]	X	X	X	X	X
Bipolar disorder or manic episode	X	X	X	X	
Confusion/disorientation	X	X			X
COPD	X		X	X	X
Cough	X		X	X	X
Delusions	X	X			X
Depression	X	X	X	X	X
Dermatitis and eczema	X		X	X	
Feeling anxious	X	X			X
GERD	X		X	X	
Irritability	X	X			X
Mood disorder	X	X	X	X	
Obsessive-compulsive disorder	X	X	X	X	X
Other respiratory disorder	X		X	X	
Overweight/Obesity	X		X	X	
Psychotic disorder[b]	X	X	X	X	
Respiratory disorders other	X		X	X	
Restlessness	X	X			X
Self-harm	X	X	X	X	X
Sleep disorder	X	X	X	X	X
Substance abuse	X		X	X	X
Type 2 Diabetes	X		X	X	

© 2026 Jaffe DH et al. *JAMA Network Open*.

Variable	Measure type		Data source		
	Covariate	Outcome	Claims	EHR structured	EHR unstructured
Uncontrolled muscle movement	X	X			X
Comorbidity index	X		X	X	
**Lifestyle characteristics**[c]					
Exercise	X				X
Alcohol use	X				X
Tobacco use	X				X
Marijuana use	X				X
**Family medical history**					
Family history of psychiatric disorder	X				X
Family history of sleep disorder	X				X
**Healthcare resource utilization**	X		X	X	
**Prescriptions medication use**	X		X		
**Asthma, asthma severity and control, and other asthma related characteristics**[d]					
Asthma severity diagnosis	X		X	X	
Shortness of breath	X				X
Snoring	X		X	X	X
Wheezing	X				X
FEV1%[e]	X			X	

[a] Attention difficulties include attention deficit hyperactivity disorder, hyperactivity, and aggression.
[b] Psychotic disorder is defined as a diagnosis of schizophrenia, schizotypal, delusional, and other non-mood psychotic disorders
[c] Lifestyle characteristics were defined as entities containing mention of the use or habits such as, practicing sports as an indicator of exercise.
[d] Healthcare resource utilization and prescription medications were also used to describe patients with asthma.
[e] Patients with an FEV1% <60% are considered as having severe asthma
Abbreviations. COPD, chronic obstructive pulmonary disease; GERD, gastro-esophageal reflux disease; EHR, electronic Health Records; FEV, forced expiratory volume; ICS, inhaled corticosteroid monotherapy;

© 2026 Jaffe DH et al. *JAMA Network Open*.

eTable 2. Baseline Demographics of Patients With Asthma in Claims Linked to Structured and Unstructured EHR Data

Variable	Overall N = 109 076		Montelukast N = 39 665		ICS N = 69 411	
	No.	%	No.	%	No.	%
**Age at treatment initiation (index)**						
**Mean (SD)**, years	28.76	20.50	30.43	20.55	27.80	20.40
**Median (Range)**, years	20	6.0 – 80.0	25	6.0 – 80.0	18	6.0 – 80.0
**Age at treatment initiation (index)**						
**6 – 11**	31 177	28.6	9 839	24.8	21 338	30.7
**12 – 17**	19 388	17.8	6 753	17.0	12 635	18.2
**18 – 29**	12 602	11.6	4 851	12.2	7 751	11.2
**30 – 39**	10 652	9.8	4 309	10.9	6 343	9.1
**40 – 49**	10 931	10.0	4 417	11.1	6 514	9.4
**50 – 59**	12 936	11.9	4 972	12.5	7 964	11.5
**60 – 69**	8 741	8.0	3 381	8.5	5 360	7.7
**70 – 80**	2 649	2.4	1 143	2.9	1 506	2.2
**Sex**						
**Female**	64 747	59.4	24 661	62.2	40 086	57.8
**Male**	44 329	40.6	15 004	37.8	29 325	42.3
**Marital Status**						
**Married or living with partner(s)**	18 964	17.4	8 197	20.7	10 767	15.5
**Not married**	82 545	75.7	28 780	72.6	53 765	77.5
**Other[a] or missing**	7 567	6.9	2 688	6.8	4 879	7.0
**Race**						
**American Indian, Alaska Native, Asian, Native Hawaiian, or Other Pacific Islander**	3 359	3.1	1 150	2.9	2 209	3.2
**Black or African American**	23 302	21.4	7 752	19.5	15 550	22.4
**White**	59 657	54.7	23 674	59.7	35 983	51.8
**Other[b]**	15 341	14.1	4 732	11.9	10 609	15.3
**Missing**	7 417	6.8	2 357	5.9	5 060	7.3
**Ethnicity**						
**Hispanic or Latino**	27 177	24.9	9 233	23.3	17 944	25.9
**Non-Hispanic or Latino[c]**	73 139	67.1	27 571	69.5	45 568	65.7
**Missing**	8 760	8.0	2 861	7.2	5 899	8.5
**Region**						
**Midwest**	14 816	13.6	5 736	14.5	9 080	13.1
**Northeast**	18 342	16.8	6 446	16.3	11 896	17.1
**South**	33 790	31.0	13 921	35.1	19 869	28.6
**West**	41 578	38.1	13 361	33.7	28 217	40.7
**Missing**	550	0.5	201	0.5	349	0.5
**Insurance Status**						
**Commercial**	29 850	27.4	12 578	31.7	17 272	24.9

© 2026 Jaffe DH et al. *JAMA Network Open*.

Variable	Overall N = 109 076		Montelukast N = 39 665		ICS N = 69 411	
	No.	%	No.	%	No.	%
Medicaid	71 993	66.0	24 028	60.6	47 965	69.1
Medicare	5 640	5.2	2 534	6.4	3 106	4.5
Other or missing	1 593	1.5	525	1.3	1 068	1.5
Source of Asthma Diagnosis						
Claims	49 758	45.6	18 698	47.1	31 060	44.8
EHR-structured	31 732	29.1	12 607	31.8	19 125	27.6
Claims and EHR-structured	27 586	25.3	8 360	21.1	19 226	27.7
Index Year						
2015	19 294	17.7	7 178	18.1	12 116	17.5
2016	23 206	21.3	8 051	20.3	15 155	21.8
2017	17 787	16.3	6 605	16.7	11 182	16.1
2018	14 588	13.4	5 872	14.8	8 716	12.6
2019	14 356	13.2	5 329	13.4	9 027	13.0
2020	9 715	8.9	3 100	7.8	6 615	9.5
2021	6 972	6.4	2 424	6.1	4 548	6.6
2022	3 158	2.9	1 106	2.8	2 052	3.0

Abbreviations. EHR, electronic Health Records; ICS, inhaled corticosteroid monotherapy; SD, standard deviation.

[a] Other includes refusal to provide marital status, multiple statuses reported, and other.

[b] Other race includes ‘other’ or multiple races reported as race.

[c] Non-Hispanic or Latino includes multiple ethnicities.

© 2026 Jaffe DH et al. *JAMA Network Open*.

eTable 3. Percentage Distribution of Components of Incident Neuropsychiatric Outcomes in Matched Unconditional Cohorts From the MOSAIC-NLP Analyses

Incident Outcomes[a]	Analysis 1 (claims data only)		Analysis 2 (claims plus structured EHR)		Analysis 3 (claims plus structured and unstructured EHR)	
	Montelukast %	ICS %	Montelukast %	ICS %	Montelukast %	ICS %
**Anxiety[b]**	49.1	49.1	49.3	49.5	39.9	39.8
**Sleep disorder[c]**	16.6	16.4	18.7	17.9	16.6	15.9
**Mood disorder - depression**	15.4	15.7	15.6	15.4	14.9	14.8
**Mood disorder - other**	13.4	12.9	9.2	9.0	8.2	7.8
**Self harm[d]**	2.8	3.0	0.3	0.4	4.6	5.2
**Psychotic disorder**	1.9	2.1	0.3	0.3	1.2	1.3
**Adult personality disorder**	0.8	0.9	1.2	1.2	0.5	0.6
**Agitation**	0.0	0.0	0.0	0.0	0.1	0.1
**Bipolar disorder or manic episode**	0.0	0.0	2.9	3.2	0.3	0.2
**Obsessive-compulsive disorder**	0.0	0.0	0.1	0.1	0.1	0.1
**Confusion/disorientation**	NA	NA	NA	NA	2.3	2.8
**Delusions**	NA	NA	NA	NA	0.1	0.1
**Attention difficulties[e]**	0.0	0.0	2.5	3.0	6.6	7.1
**Hallucinations**	NA	NA	NA	NA	2.8	2.2
**Irritability**	NA	NA	NA	NA	0.6	0.6
**Memory problems**	NA	NA	NA	NA	0.3	0.2
**Restlessness**	NA	NA	NA	NA	0.2	0.2
**Tremor/shakiness**	NA	NA	NA	NA	0.0	0.0
**Uncontrolled muscle movement**	NA	NA	NA	NA	0.9	0.9

Abbreviations. EHR, electronic health record, ICS, inhaled corticosteroids, MOSAIC-NLP, Multi-source Observational Safety study for Advanced Information Classification using Natural Language Processing, NA, not applicable.

[a] Outcomes were identified using only claims data (Analysis 1), claims + EHR-structured data (Analysis 2), and claims + EHR-structured data + EHR-unstructured data (Analysis 3). Not applicable (NA) represents outcomes not collected as part of the analysis.

[b] Anxiety includes diagnoses or mention of anxiety disorder, anxiety, and feeling anxious.

[c] Sleep disorders include insomnia, hypersomnia, circadian rhythm disorder, parasomnia, movement disorder, other undefined sleep disorders, or treatment for sleep disorders

[d] Self-harm includes self-harm, self-harm ideation, suicide attempt, and suicide ideation.

[e] Attention difficulties include attention deficit hyperactivity disorder, hyperactivity, and aggression.

© 2026 Jaffe DH et al. *JAMA Network Open*.

eFigure 1. Study Design

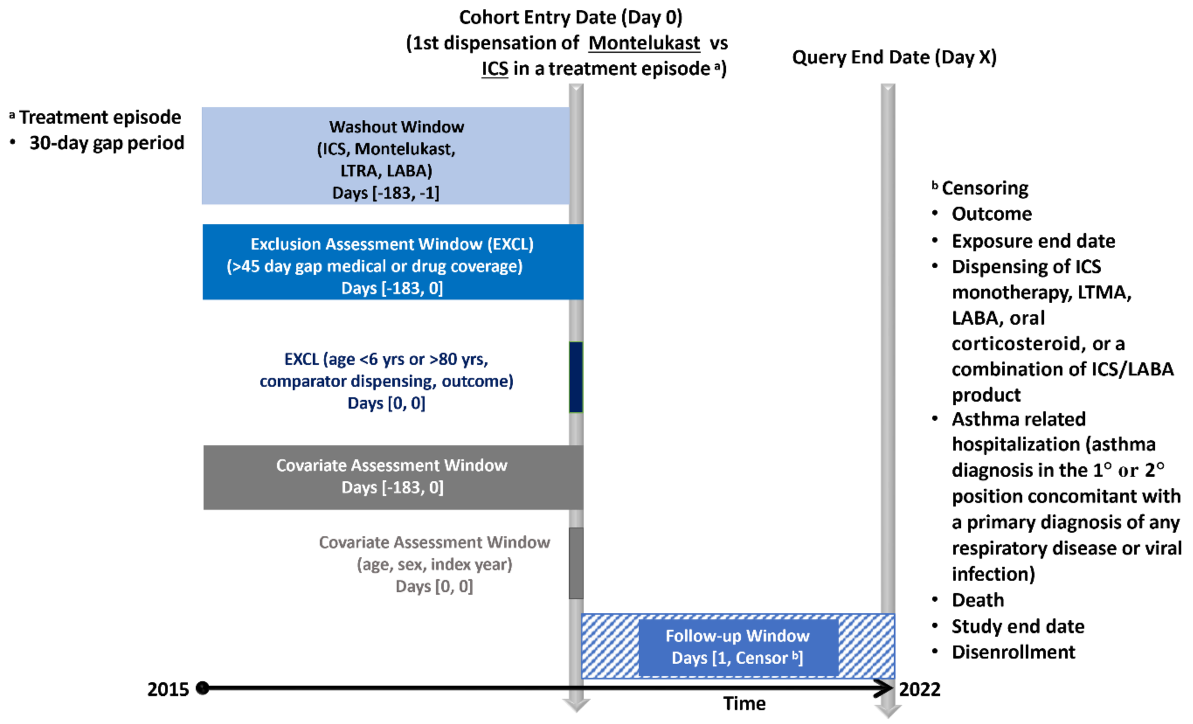

Abbreviations. EXCL, exclusion assessment window; ICS, inhaled corticosteroid; LABA, long-acting beta agonist; LTRA, leukotriene receptor antagonist

© 2026 Jaffe DH et al. *JAMA Network Open*.

eReferences

1. Ehwerhemuepha L, Carlson K, Moog R, et al. Cerner real-world data (CRWD) - A de-identified multicenter electronic health records database. Data Brief. 2022;42:108120. doi:10.1016/j.dib.2022.108120
2. Oracle Health. Real-World Data. Accessed September 8, 2024. https://www.oracle.com/health/population-health/real-world-data/
3. Datavant. Mortality Data in Healthcare. Accessed November 5, 2025. https://www.datavant.com/white-papers/mortality-data-in-healthcare-analytics
4. Berliner E, Jaffe D, Toh D, et al. Inclusion of Semi-Structured and Unstructured Electronic Health Record (EHR) Data in Confounding Adjustment and Outcome Ascertainment | Sentinel Initiative. Accessed March 31, 2024. https://www.sentinelinitiative.org/methods-data-tools/methods/inclusion-semi-structured-and-unstructured-electronic-health-record-ehr
5. Jaffe D, Balkaran B, Desai, R, Dutcher SK. Natural Language Processing in Pharmacoepidemiology: Lessons from the Multi-Source Observational Safety study for Advanced Information Classification Using NLP (MOSAIC-NLP). Published online Aug 26, 2024. Accessed July 27, 2025. https://www.sentinelinitiative.org/news-events/publications-presentations/natural-language-processing-pharmacoepidemiology-lessons
6. Zhang E, Levin AM, Williams LK. How does race and ethnicity effect the precision treatment of asthma? Expert Rev Precis Med Drug Dev. 2019;4(6):337-356. doi: 10.1080/23808993.2019.1690396.
7. US Centers for Disease Control and Prevention. PHIN Vocabulary. Accessed November 3, 2025. https://www.cdc.gov/phin/php/vocabulary/index.html.

© 2026 Jaffe DH et al. *JAMA Network Open*.
