# Supplementary material for: Use of Clinical Notes to Assess Neuropsychiatric Events After Montelukast Initiation
Source: JAMA Netw Open. 2026 Feb 12;9(2):e2558433. doi: 10.1001/jamanetworkopen.2025.58433 (PMC12902883; doi:10.1001/jamanetworkopen.2025.58433)
Supplement: Supplement 2. — Data Sharing Statement [file jamanetwopen-e2558433-s002.pdf]

Data Sharing Statement

Jaffe. Leveraging Clinical Notes in a Safety Study of Neuropsychiatric Events After Montelukast Initiation. *JAMA Netw Open*. Published February 12, 2026.
doi:10.1001/jamanetworkopen.2025.58433

Data
Data available: No

Additional Information
Explanation for why data not available: Oracle Real World data is proprietary and therefore cannot be shared, but that we have shared the NLP annotation guideline and all code for the project so that the results may be replicated in EHR/claims and clinical notes data held by other researchers. As part of this project, we demonstrated the transportability of the code (see article text).
